# Evaluating cord blood S100B protein concentration as a potential biomarker associated with fetal hypoxia in late fetal growth restriction: A prospective cohort study protocol

**DOI:** 10.1371/journal.pone.0355156

**Published:** 2026-08-05

**Authors:** Agnieszka Drozdowska-Szymczak, Sabina Adrianna Łukawska, Natalia Mazanowska, Beata Broniarek-Samson, Jakub Zdulski, Ewa Mierzejewska, Tadeusz Issat, Paweł Krajewski

**Affiliations:** 1 Department of Neonatology and Neonatal Intensive Care, Institute of Mother and Child, Warsaw, Poland; 2 Department of Obstetrics and Gynecology, Institute of Mother and Child, Warsaw, Poland; 3 Central Laboratory, Institute of Mother and Child, Warsaw, Poland; 4 Department of Epidemiology and Biostatistics, Institute of Mother and Child, Warsaw, Poland; Imperial College London, UNITED KINGDOM OF GREAT BRITAIN AND NORTHERN IRELAND

## Abstract

**Introduction:**

S100B protein is a biomarker associated with central nervous system (CNS) injury following hypoxic-ischemic events. Measurement of S100B levels in umbilical cord blood may provide a noninvasive means of identifying newborns exposed to perinatal hypoxia related to fetal growth restriction (FGR) immediately after birth. Early detection of infants at increased risk of hypoxia-related CNS injury could facilitate closer surveillance, prompt diagnostic evaluation, and timely implementation of appropriate interventions, potentially improving long-term neurodevelopmental outcomes.

**Methods and Analysis:**

This study investigates two groups of full-term pregnancies: a study group with prenatally diagnosed late FGR, and a control group with normal fetal growth. Following delivery, cord blood samples from both groups will be analyzed for S100B protein concentrations, pH, base excess (BE), and lactate levels. Additionally, fetal blood flow parameters in the umbilical artery (UA), uterine arteries (UtA), ductus venosus (DV), and middle cerebral artery (MCA) will be assessed via ultrasound within 48 hours before delivery. This study aims to compare S100B protein concentrations in umbilical cord blood between the two groups and evaluate its correlations with fetal Doppler parameters, pH, BE, and lactate levels in cord blood gas analysis. Additionally, it will evaluate the potential utility of S100B protein concentration as a biomarker associated with perinatal hypoxic stress in FGR-affected neonates.

**Ethics and Dissemination:**

The study received approval from the Bioethics Committee of the Institute of Mother and Child in Warsaw (Decision No. 13/2024, dated 21 March 2024). The findings will be submitted for publication in peer-reviewed journals specializing in neonatology, pediatrics, obstetrics, and perinatology. Additionally, abstracts will be submitted to relevant national and international conferences.

**Trial registration:**

The study is registered at ClinicalTrials.gov (Identifier: NCT06893926).

## Introduction

Fetal hypoxia-ischemia (HI) presents a major challenge in perinatology due to its potential for causing severe complications in the child. Hypoxemia can result in brain damage, which may lead to conditions such as cerebral palsy, epilepsy, delayed psychomotor development, intellectual disability, and cognitive impairment [1–3].

Currently, the diagnosis of neonatal HI relies on a combination of prenatal and postnatal data. Abnormal UA and MCA blood flow parameters detected prenatally may indicate a risk of fetal HI. Postnatally, the diagnosis is based on neurological examination, laboratory tests, neurophysiological assessments, and imaging studies (such as ultrasound and MRI), along with ultrasonographic evaluation of blood flow parameters in the MCA and anterior cerebral artery (ACA) [1,3]. Despite these methods, some infants with CNS damage may not show immediate symptoms or abnormalities in additional tests after birth, highlighting the need for improved early identification strategies. Cord blood biomarkers, such as the S100B, are therefore of significant interest [1–11].

S100B is a calcium-binding regulatory protein found in the cytoplasm of brain cells. In cases of CNS damage, such as hypoxia, S100B is released into the bloodstream, resulting in elevated levels in cerebrospinal fluid and umbilical cord blood [2,8,12]. This allows for the noninvasive assessment of the biomarker’s concentration immediately after birth. Due to its short half-life, S100B facilitates the recognition and monitoring of CNS damage progression, with its concentration correlating with the severity of hypoxia. Other factors that can elevate S100B levels in a newborn’s bloodstream and cord blood include chorioamnionitis, preterm birth, prolonged vaginal labor (lasting over 15 hours), and operative delivery [5,13–17]. Conversely, significantly lower S100B levels have been observed in infants born to mothers treated with selective serotonin reuptake inhibitors (SSRIs) [18].

Higher concentrations of S100B protein have been reported in cases of chronic hypoxia, particularly in newborns diagnosed with FGR or small for gestational age (SGA), where birth weight is below the 10th percentile. However, these findings are sometimes contradictory [1–3,6]. Among neonates with FGR, significantly higher S100B concentrations have been observed in the umbilical vessels of those with abnormal prenatal blood flow in the UA compared to those with normal flow parameters [2,6]. In the FGR group, elevated S100B protein levels have also been linked to an increased risk of necrotizing enterocolitis (NEC), the need for intubation, mortality, and, according to some studies, the occurrence of intraventricular hemorrhage (IVH) [5,6]. In growth-restricted neonates, there is a noted correlation between neurological developmental disorders and blood flow parameters in the fetal MCA. However, the impact of the severity of blood flow impairment in the fetal brain on S100B concentration has not yet been studied [19].

Assessing cord blood S100B protein concentration may enable the early identification of newborns at high risk for abnormal development, even when standard biochemical or clinical parameters and neurological assessments reveal no abnormalities.

Furthermore, identifying a child at risk on the first day of life through noninvasive methods could enable early planning for systematic pediatric and physiotherapy care. This proactive approach would allow for the timely initiation of rehabilitation, potentially improving neurodevelopmental outcomes in infants affected by intrauterine growth disorders.

## Methods and analysis

The protocol was prepared with consideration of STROBE recommendations applicable to observational cohort studies [20,21] and registered at ClinicalTrials.gov (NCT06893926) on March 25, 2025 (https://clinicaltrials.gov/study/NCT06893926).

### Objectives

The study aims to:

1. Determine whether the cord blood S100B protein concentration is significantly higher in neonates with prenatally diagnosed FGR compared to controls.
2. Evaluate the correlation between S100B protein concentration and fetal blood flow parameters in the UA, UtA, DV, and MCA, as assessed by ultrasound performed within 48 hours before delivery.
3. Evaluate the discriminatory performance of S100B concentration in relation to perinatal markers associated with fetal hypoxia (pH, BE, and lactate levels in umbilical artery blood).

### Trial design

This single-center, prospective cohort study aims to assess the effectiveness of S100B protein concentration as a potential biomarker associated with perinatal hypoxic stress in neonates with FGR, compared to a control group without growth disorders.

### Study setting

The study is being conducted at the Institute of Mother and Child in Warsaw, specifically within the Department of Obstetrics and Gynecology and the Department of Neonatology and Neonatal Intensive Care, both of which function as tertiary referral units. The center manages approximately 50 full-term newborns diagnosed with FGR annually. Recruitment commenced in June 2024 and was initially projected to continue for approximately 18 months, through December 2025, with the actual duration dependent on the number of FGR patients enrolled during that period. Complete data collection is expected to be finalized within three months of the end of recruitment (March 2026), with initial results anticipated in June 2026. At the time of protocol publication, the authors anticipated potential minor delays in patient recruitment due to a lower-than-expected incidence of pregnancies complicated by FGR at the study center, as well as a higher-than-anticipated number of exclusions. As of September 2025, approximately two-thirds of the targeted patient population have been enrolled in both the study and control groups.

### Definitions

Fenton’s growth charts are used to assess birth weight percentiles [22]. The criteria for diagnosing FGR follow the 2020 recommendations of the Polish Society of Gynecologists and Obstetricians for late-onset growth restriction, which occurs after the 32nd week of pregnancy [23]. These guidelines are based on the Delphi Criteria for diagnosing early and late FGR [24].

Late FGR is diagnosed if any of the following criteria are detected during routine fetal ultrasound:

- Abdominal circumference (AC) below the 3rd percentile for the given gestational age.
- Estimated fetal weight (EFW) below the 3rd percentile for the given gestational age.

OR

- EFW or AC below the 10th percentile, along with at least one of the following:
  - Growth retardation by the 50th percentile on the growth curve.
  - Cerebroplacental index (CPR), defined as the ratio of the pulsatility indices (PI) in the MCA and UA, below the 5th percentile for the given gestational age.
  - UA PI above the 95th percentile for the given gestational age.

### Eligibility criteria

#### Study group – Inclusion criteria.

1. Women with a full-term, singleton pregnancy (≥37 + 0/7 weeks of gestation).
2. Pregnancy complicated by late-onset FGR.

#### Study group – Exclusion criteria.

1. Antenatal (at recruitment):
  - Maternal conditions that may affect the blood flow in placental vessels, including smoking, use of illicit stimulant substances, or pregestational diabetes.
  - Maternal depression requiring pharmacological treatment (e.g., SSRIs).
2. Intrapartum:
  - Factors indicating a possible intrauterine infection, such as amniotic fluid leakage for more than 15 hours, spontaneous preterm labor, diagnosed intrauterine infection, or maternal symptoms of infection.
  - Prolonged labor lasting more than 15 hours.

Given the data on increased S100B level in labor exceeding 15 hours, this duration of amniotic fluid leakage was set as the cutoff point in the exclusion criteria [5,17].

#### Control Group – Inclusion criteria.

1. Women with a full-term, singleton pregnancy (≥37 + 0/7 weeks of gestation).
2. Pregnancy not complicated by FGR.

#### Control Group – Exclusion criteria.

1. Antenatal (at recruitment):
  - Maternal conditions that may affect placental blood flow, such as smoking, use of illicit stimulant substances, pregestational diabetes, or chronic hypertension.
  - Maternal depression requiring pharmacological treatment (e.g., SSRIs).
  - Risk factors for intrauterine HI, including abnormal fetal blood flow parameters on ultrasound, abnormal CTG recordings, or the need for intrauterine transfusion (IUT).
2. Intrapartum:
  - Indicators of possible intrauterine infection, such as amniotic fluid leakage for more than 15 hours, spontaneous preterm delivery, diagnosed intrauterine infection, or maternal symptoms of infection.
  - Risk factors for perinatal HI.
  - Prolonged labor lasting more than 15 hours (counted from the onset of regular uterine contractions).
  - Birth weight below the 10th percentile or above the 90th percentile.
  - Apgar score less than 8 at the 1st, 3rd, 5th, or 10th minute of life.
  - Abnormal umbilical cord blood gas analysis results, defined as pH < 7.15 or BE < −9.3 mmol/l.
3. Postnatal:
  - Neonatal anemia requiring a top-up transfusion within the first 24 hours of life.

The exclusion criteria for the study and control groups differ based on the principle that the control group should consist of healthy neonates without risk factors for HI. In contrast, the study group includes infants diagnosed with FGR, who inherently have a higher risk of complications. These complications, such as a low Apgar score, abnormal umbilical cord blood gas analysis parameters, or an abnormal CTG recording, are precisely the factors that serve as exclusion criteria for the control group. This approach ensures that the control group provides a valid baseline for comparison against the at-risk study group.

In patients with a singleton, full-term pregnancy who present to the labor and delivery unit and meet the inclusion criteria, without meeting the antenatal exclusion criteria (except for those related to FGR), informed written consent for testing is obtained prior to delivery. The mother is also informed that if any exclusion criteria are identified during or after delivery, the child will be disqualified from further participation in the study.

To account for the potential influence of the mode of delivery (vaginal delivery [VD] or cesarean section [CS]) on S100B protein concentration, the study aims to balance the proportion of neonates delivered by each method between the study and control groups. As recruitment progresses, the number of pregnancies delivered by VD and CS in the study group will be monitored. Recruitment in the control group will be adjusted based on the mode of delivery to ensure that the percentage of CS is similar in both groups. This approach aims to minimize any confounding effects of the delivery method on the study outcomes.

### Study procedures and data collection

Data will be collected through cord blood samples, ultrasound measurements, and clinical assessments.

For all patients who consent to participate in the study, an ultrasound examination will be performed within 48 hours before delivery. This ultrasound assessment includes:

a. Measurement of the fetus’s anthropometric dimensions and estimation of fetal weight.
b. Evaluation of blood flow using the pulsatility index (PI) in the UA, UtA, DV, and MCA.

After birth, in both the study and control groups:

1. A 1.5 ml blood sample will be collected from the clamped part of the umbilical cord:
  a. Immediate analysis: 0.5 ml of the cord blood will be sent immediately to the laboratory to determine pH, BE, and lactate levels in the umbilical cord blood analysis.
  b. S100B protein analysis: The remaining 1 ml of cord blood will be placed in a labeled tube (including the mother’s name, child’s gender, date of birth, and date of collection) and sent to the laboratory for centrifugation. Serum samples will be frozen at −20°C and stored. Once approximately 80 samples are collected, the serum will be thawed and analyzed for S100B protein concentration. Any remaining material after laboratory processing will be properly disposed of.
2. Data will be collected on gestational age, duration, and mode of delivery, along with any complications encountered during delivery. Additionally, details regarding the newborn’s condition after birth will be recorded, including gender, Apgar score, and anthropometric measurements. Observations made during hospitalization will also be documented, with particular attention to any concerning symptoms related to the CNS, such as muscle tone abnormalities or seizures.
3. A transfontanelle ultrasound examination will be performed to assess for any abnormalities in the newborn.

Study participation does not alter standard hospitalization procedures or duration. Furthermore, collecting a blood sample for the planned tests will not interfere with cord blood collection if the mother intends to bank it.

### Criteria for discontinuing participation

If a full ultrasound flow assessment becomes unfeasible due to technical issues, advanced labor, or urgent delivery indications, it will be halted. However, any partial data collected will still be analyzed.

If an umbilical cord blood sample cannot be collected after birth, the patient will be excluded from the study. Nevertheless, data from excluded patients will be retained to support further analysis and ensure a comprehensive statistical evaluation.

In cases where consent is withdrawn, any data collected up to that point will be retained and used in the study.

### Compliance

Before the study commenced, a meeting was held to familiarize the researchers and staff with the study protocol. The protocol was presented in detail, including the rationale behind the study. Additionally, schematic instructions were provided, covering patient qualification, the required tests for qualification, and the intervention procedures.

The medical records of participating patients are clearly labeled to ensure adherence to the study protocol. Each patient’s medical record includes a schematic outlining the inclusion and exclusion criteria.

### Explanation for the choice of comparators

Biomarkers associated with the risk of hypoxia-related CNS injury should meet several key criteria:

- They should be detectable in easily accessible fluids such as blood, amniotic fluid, or urine, allowing for straightforward sample collection.
- They should identify patients at risk before symptoms emerge or clinical examinations, or other tests (e.g., ultrasound, MRI, or EEG) reveal any abnormalities.
- They should provide a quantitative measure of the severity of perinatal hypoxic stress.
- The tests should be simple, quick, cost-effective, and characterized by repeatability, sensitivity, and specificity [25].

To date, few markers have been thoroughly evaluated for each of these criteria. However, the S100B protein shows promise as a potential marker. It can be detected in blood, saliva, urine, and amniotic fluid, potentially allowing for the early identification of neonates at risk of adverse neurodevelopmental outcomes in a relatively quick and inexpensive manner. Nonetheless, additional prospective studies are needed.

### Outcome measures

The study’s primary endpoint will be the concentration of S100B protein measured from a cord blood sample taken after birth in both the study and control groups.

Additional endpoints will include:

- The potential correlation between S100B concentration and pH, BE, and lactate levels, as determined in the cord blood gas analysis in both the study and control groups.
- The possible relationship between cord blood S100B protein concentration and blood flow parameters (PI) in the UA, UtA, DV, and MCA, as assessed by fetal ultrasound performed within 48 hours before delivery.

### Participant timeline

**Fig 1:**
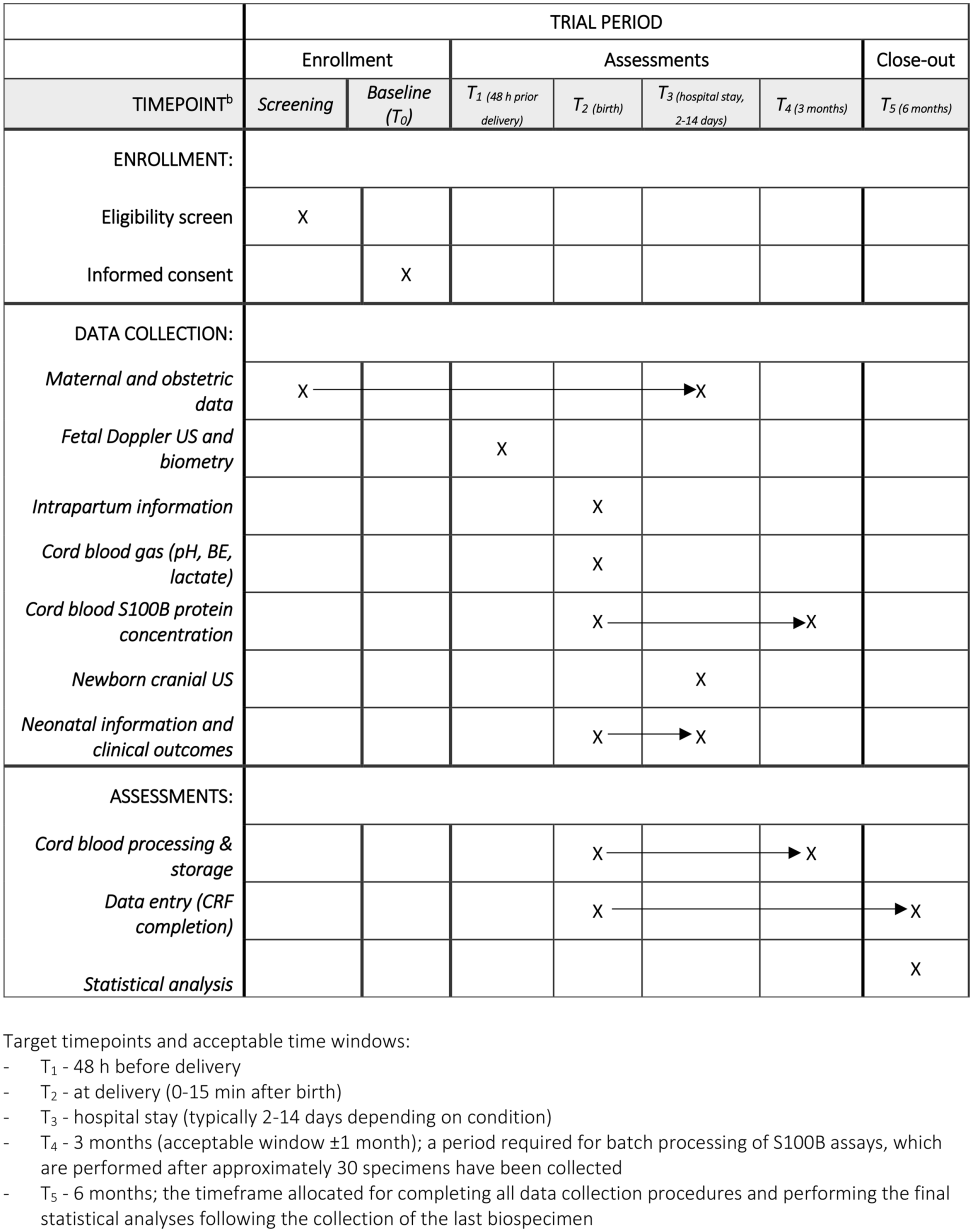
Participant timeline according to the SPIRIT 2025 guidelines. Schedule of enrollment, data collection, assessments, and follow-up procedures in the prospective cohort study evaluating cord blood S100B protein concentration as a potential biomarker associated with fetal hypoxia in late fetal growth restriction. The timeline presents study procedures from screening and enrollment through baseline assessment, perinatal data collection, neonatal assessments, sample processing, data entry, and final statistical analysis. T0 indicates baseline assessment; T1–T5 represent subsequent study time points (T1: 48 hours after delivery, T2: birth, T3: hospital discharge or 2–14 days, T4: 3 months, T5: 6 months). X indicates the time point of assessment or data collection; arrows indicate ongoing procedures or follow-up periods.

For the primary endpoint data acquisition, the study period includes the fetal ultrasound examination conducted 48 hours before delivery and the delivery process itself. This encompasses umbilical cord clamping, collecting cord blood from the severed portion of the umbilical cord, and recording anthropometric measurements. Additional data that may lead to patient exclusion (e.g., diagnosis of intrauterine infection or neonatal anemia requiring a top-up transfusion in the control group) or that could impact the study results (e.g., transfontanelle ultrasound findings or abnormal neurological symptoms) will be collected until the child is discharged from the hospital. The overall study design and timeline are presented in Fig 1.

### Sample size

The estimated minimum total sample size for the study is 120 patients, with 60 participants in each group. The planned sample size was determined for a two-sided comparison between two independent groups, assuming a significance level of 0.05, statistical power of 80%, and equal group sizes. The variability of the primary biomarker (S100B) was estimated based on data from a previously published reference study [12], which reported median values together with the 10th and 90th percentiles. As standard deviations were not provided, dispersion was approximated from the reported percentiles under an approximate normality assumption, following established methods for recovering variability from limited summary statistics [26,27]. In the reference study, the observed difference between group medians was 1.15. However, since group allocation in that study was performed retrospectively on the basis of cranial imaging findings (brain injury vs. no brain injury), a conservative approach was adopted in the present sample size estimation to minimize the risk of effect size overestimation. Therefore, the target detectable difference was defined as 50% of the previously reported median difference.

Based on these assumptions, the estimated required sample size was approximately 55 participants per group. Additionally, considering that up to 10% of collected samples could be non-diagnostic or otherwise non-evaluable, the planned recruitment target was increased to 60 participants per group (120 participants in total). Sample size calculations were performed using standard power analysis procedures implemented in G*Power software (version 3.1.9.4).

### Sequence generation

Participants are assigned to the study group based on a diagnosis of FGR from routine fetal ultrasound and the absence of exclusion criteria. The control group comprises participants based on gestational age (≥37 + 0/7 weeks), no diagnosis of FGR on ultrasound, and the absence of exclusion criteria for this group.

### Data collection

Patients’ data are collected using a password-protected electronic form, with access restricted to the researchers involved in the study.

The researchers are responsible for conducting blood tests on the umbilical cord blood and performing both fetal and postnatal transfontanelle ultrasound examinations.

### Statistical methods

Descriptive statistics, including percentages for categorical variables and mean values, standard deviations, medians, and interquartile ranges for continuous variables, will be used to summarize the data. The Kolmogorov-Smirnov test, along with graphical analysis, will be employed to assess whether continuous variables conform to a normal distribution.

For analyzing the relationship between a quantitative variable and a qualitative variable (e.g., comparing S100B levels between the study and control groups), the Student’s t-test for independent samples will be used if the continuous variable is normally distributed. If the continuous variable does not meet the assumptions of normality, the Mann-Whitney U test will be applied instead.

To test hypotheses about the relationships between two continuous variables (e.g., S100B protein concentration versus flow rates), while accounting for confounding factors such as pregnancy duration and mode of delivery, either multivariate quantile regression or multivariate linear regression will be employed, depending on the distribution of the outcome variable.

### Patient and public involvement

Patients and the public were not involved in the design of the study.

### Ethics and dissemination

The study has received approval from the Bioethics Committee of the Institute of Mother and Child in Warsaw (Decision No. 13/2024, dated 21 March 2024). Any significant modifications to the protocol, including changes to the study’s purpose, design, population, inclusion or exclusion criteria, sample size, or procedures, will be documented as formal amendments in the study protocol and reported to the Bioethics Committee of the Institute of Mother and Child in Warsaw.

Participant recruitment is conducted by a designated member of the research team. Potential participants receive both oral and written information about the study and are allowed to ask questions. Written informed consent is obtained from each participant before enrollment.

All data collected from individual participants will be de-identified prior to analysis and data sharing. The de-identified dataset, together with the full study protocol, will be made available without time restrictions to researchers who submit a methodologically sound proposal, following publication of the study results.

Data monitoring is performed by the Science Department of the Institute of Mother and Child in Warsaw, the authors’ institutional affiliation, which receives periodic reports on study progress from the research team.

The study’s results will be published in a peer-reviewed journal related to neonatology, pediatrics, obstetrics, or perinatology.

## Study strengths, limitations and future directions

1. To address the knowledge gap, the study aims to answer the following questions:
  - Is cord blood S100B protein concentration significantly higher in infants with prenatally diagnosed late FGR, a condition associated with increased risk of hypoxic CNS injury?
  - Does the concentration of S100B protein in umbilical cord blood correlate with blood flow parameters in the UA, UtA, DV, and MCA, as assessed by ultrasound within 48 hours before delivery?
  - Is there an association between S100B protein concentration and pH, BE, and lactate levels in cord blood, in the context of evaluating perinatal hypoxic stress?
2. The study procedures are straightforward and confined to a specific time point, which facilitates the accurate implementation of the study protocol.
3. The study evaluates only short-term outcomes and does not account for the long-term neurological development of the children.
4. In cases of urgent indications for delivery or an advanced stage of labor at admission, performing a reliable Doppler flow assessment may not be feasible.
5. In some instances, collecting the necessary volume of cord blood for testing may be difficult due to technical problems.
6. Due to numerous exclusion criteria in both the control and study groups, which may arise after informed consent is obtained, some patients may be disqualified during the study.

The present study may serve as a starting point for further investigations into the usefulness of S100B protein as a biomarker associated with the risk of hypoxia-related CNS injury in neonates with late FGR. Future research should consider extending the diagnostic approach to include long-term neurological follow-up, as well as additional assessment of cerebral blood flow using postnatal ultrasonography and monitoring of cerebral bioelectrical activity with amplitude-integrated electroencephalography (aEEG). Such an approach could enable evaluation of the relationship between perinatal S100B protein concentrations and subsequent neurodevelopmental outcomes in children with FGR.

A limitation of the present study is its short-term design and the lack of long-term neurological follow-up of the included neonates. Further prospective studies involving extended clinical follow-up are necessary to determine whether elevated S100B protein concentrations are associated with permanent CNS injury or later neurodevelopmental disorders. Therefore, the present study primarily enables assessment of associations between FGR, markers of fetal hypoxia, and S100B concentrations rather than definitive evaluation of long-term neurodevelopmental outcomes.

## Supporting information

S1 File SPIRIT 2025 checklist. (DOCX)

S2 File SPIRIT 2025 participant timeline. (DOCX)

S3 File STROBE checklist for cohort studies. (DOC)
