# Supplementary material for: Evaluating cord blood S100B protein concentration as a potential biomarker associated with fetal hypoxia in late fetal growth restriction: A prospective cohort study protocol
Source: PLoS One. 2026 Aug 5;21(8):e0355156. doi: 10.1371/journal.pone.0355156 (PMC13440886; doi:10.1371/journal.pone.0355156)
Supplement: Supplementary file 2 [file pone.0355156.s002.docx]

|  | **TRIAL PERIOD** | | | | | | | |
| --- | --- | --- | --- | --- | --- | --- | --- | --- |
|  | **Enrollment** | | | **Assessments** | | | | **Close-out** |
| **TIMEPOINT^b^** | ***Screening*** | ***Baseline (T_0_)*** | | ***T_1_ _(48 h prior delivery)_*** | ***T_2_ _(birth)_*** | ***T_3 (hospital stay, 2-14 days)_*** | ***T_4 (3 months)_*** | ***T_5 (6 months)_*** |
| **ENROLLMENT:** |  | | | | | | | |
| **Eligibility screen** | X | |  |  |  |  |  |  |
| **Informed consent** |  | | X |  |  |  |  |  |
| **DATA COLLECTION:** |  | | | | | | | |
| ***Maternal and obstetric data*** | X | |  |  |  | X |  |  |
| ***Fetal Doppler US and biometry*** |  | |  | X |  |  |  |  |
| ***Intrapartum information*** |  | |  |  | X |  |  |  |
| ***Cord blood gas (pH, BE, lactate)*** |  | |  |  | X |  |  |  |
| ***Cord blood S100B protein concentration*** |  | |  |  | X |  | X |  |
| ***Newborn cranial US*** |  | |  |  |  | X |  |  |
| ***Neonatal information and clinical outcomes*** |  | |  |  | X | X |  |  |
| **ASSESSMENTS:** |  | | | | | | | |
| ***Cord blood processing & storage*** |  | |  |  | X |  | X |  |
| ***Data entry (CRF completion)*** |  | |  |  | X |  |  | X |
| ***Statistical analysis*** |  | |  |  |  |  |  | X |

Target timepoints and acceptable time windows:

- **T_1_ -** 48 h before delivery
- **T_2_ - a**t delivery (0-15 min after birth)
- **T_3_ - h**ospital stay (typically 2-14 days depending on condition)
- **T_4_ -** 3 months (acceptable window ±1 month); a period required for batch processing of S100B assays, which are performed after approximately 30 specimens have been collected
- T_5_ - 6 months; the timeframe allocated for completing all data collection procedures and performing the final statistical analyses following the collection of the last biospecimen
